# Non‐Kinetic Effects Convolute Activity and Tafel Analysis for the Alkaline Oxygen Evolution Reaction on NiFeOOH Electrocatalysts

**DOI:** 10.1002/anie.202216477

**Published:** 2023-01-10

**Authors:** Onno van der Heijden, Sunghak Park, Jordy J. J. Eggebeen, Marc T. M. Koper

**Affiliations:** ^1^ Leiden Institute of Chemistry Leiden University Einsteinweg 55 2333 CC Leiden The Netherlands

**Keywords:** Alkaline Oxygen Evolution Reaction, Bubble Formation, Loading, Sonication, Tafel Slope Analysis

## Abstract

A large variety of nickel‐based catalysts has been investigated for the oxygen evolution reaction (OER) in alkaline media. However, their reported activity, as well as Tafel slope values, vary greatly. To understand this variation, we studied electrodeposited Ni_80_Fe_20_OOH catalysts with different loadings at varying rotation rates, hydroxide concentrations, with or without sonication. We show that, at low current density (<5 mA cm^−2^), the Tafel slope value is ≈30 mV dec^−1^ for Ni_80_Fe_20_OOH. At higher polarization, the Tafel slope continuously increases and is dependent on rotation rate, loading, hydroxide concentration and sonication. These Tafel slope values are convoluted by non‐kinetic effects, such as bubbles, potential‐dependent changes in ohmic resistance and (internal) OH^−^ gradients. As best practise, we suggest that Tafel slopes should be plotted vs. current or potential. In such a plot, it can be appreciated if there is a kinetic Tafel slope or if the observed Tafel slope is influenced by non‐kinetic effects.

## Graphical abstract

We studied NiFeOOH catalysts for the alkaline oxygen evolution reaction (OER) with different loading, varying rotation rate, hydroxide concentration and with and without sonication. We show that the Tafel slope initially is ≈30 mV dec^−1^ for NiFeOOH in 0.05–0.2 M KOH, and at higher polarization the Tafel slope continually increases due to bubbles, potential‐dependent changes in ohmic resistance, and (internal) OH^−^ gradients.

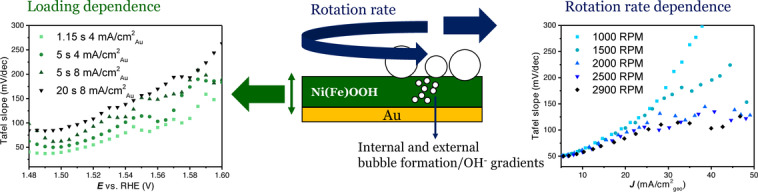

## Introduction

The oxygen evolution reaction (OER) is a much studied and important reaction for the greenification of the chemical industry, as it is the complementary process for both hydrogen evolution and CO_2_ reduction. This reaction can be performed in both acidic and basic conditions, with acidic conditions having the advantage of being compatible with the more efficient proton exchange membrane (PEM) electrolyser. However, iridium based oxides are preferred as catalysts due to their unique stability in harsh acidic conditions, but the scarcity and cost of iridium limits its large‐scale application.^[1]^ For alkaline conditions, nickel based catalysts are often preferred as they are relatively abundant, cheap and show good activity. In the past decade it has become increasingly clear that the nickel catalyst itself is not so active, but the iron impurities present in the electrolyte that adsorb and deposit in the catalyst result in strongly improved OER activity.^[2]^ Iron can also be co‐deposited with nickel, increasing the activity further.^[3]^ Usually, these OER catalyst layers are not very well defined and they are prone to change during cycling. For NiFe based materials, this usually results in a γ‐NiFeOOH layered double hydroxide (LDH) as the active phase.^[4]^

In literature, a large multitude of nickel‐based catalysts has been prepared and compared. However, benchmarking OER catalysts is a challenge, as it is difficult to determine an accurate electrochemical surface area (ECSA) and an evaluation of intrinsic activity requires an accurate ECSA rather than just the geometric area.^[5]^ Therefore, the overpotential at 10 mA per geometric cm^2^ (10 mA cm^−2^_geo_) is often used as an activity benchmark.^[6]^ As a result, high geometric activity is often a consequence of a mere increase in ECSA and not (always) due to an enhancement of intrinsic activity.^[7]^

Another important activity metric that is often reported is the Tafel slope. The Tafel slope describes how the current responds to changes in potential, and is usually reported in mV dec^−1^. An advantage of using the Tafel slope as a metric is that the Tafel slope value does not depend on the surface area, which is, as mentioned, notoriously difficult to determine. Since low Tafel slopes lead to a rapidly increasing activity with potential, low Tafel slopes are often taken as a characteristic of a highly active catalyst. Moreover, it is well known that mechanistic information can be extracted from certain “cardinal” values of the Tafel slope when certain conditions are fulfilled.^[8]^ These conditions include the absence of mass transfer limitations,^[9]^ fully compensated for ohmic resistances^[10]^ and a potential‐ and time‐independent accessible surface area. For OER, Tafel slopes are often reported by fitting (multiple) linear regions in the Tafel slope plot. Two linear regions are often observed; the second higher current density region with a higher Tafel slope is often ascribed to a change of the rate determining step,^[11]^ or to pseudocapacitive charging.^[12]^ The magnitude of these reported (single or double) Tafel slope values varies strongly between papers, while the elemental composition of the catalysts is often the same or very similar, as tabulated in multiple reviews.^[13]^ For NiFe LDHs of different loading, obtained with different deposition methods and with different promoters, the Tafel slope values tabulated in these reviews range from 27 to 71 mV dec^−1^ in similar electrolytes. For the interpretation of reported catalyst activity metrics it is essential to understand why this discrepancy exists.

To shine light on this issue of the strong variation in observed Tafel slopes, we study here the OER Tafel slope on electrodeposited Ni_80_Fe_20_OOH layers with different loadings on a rotating disk electrode (RDE), over a wide potential range, at different rotation rates (1000–2900 RPM), and with or without sonication in 0.05–0.2 M KOH. Rotation and sonication are used primarily to suppress concentration gradients and facilitate bubble detachment. To follow the evolution of the Tafel slope, it is plotted over a small range (5 or 20 mV) vs. the average current or potential. We find that, at low current density (<5 mA cm^−2^), the Tafel slope value is ≈30 mV dec^−1^ at all studied pH values, whereas at high current density there is an increase in Tafel slope value that is strongly dependent on bubble removal efforts, such as rotation and sonication, and on the hydroxide concentration. This increase in Tafel slope is most likely caused by both internal and external bubble formation blocking surface area, a resulting increase in ohmic resistance, and (internal) OH^−^ concentration gradients. Catalyst layers of different loadings show divergent Tafel slope values for the same potential regions, which indicates how loading strongly affects the reported metrics,^[14]^ even though the assumption for the Tafel slope is that it does not change with loading. Therefore, we only consider the converging Tafel slope value of 30 mV dec^−1^ at low current density as a “fundamental” kinetic Tafel slope value, while the other (higher) Tafel slope values at higher current density are convoluted by non‐kinetic effects.

As best practise, we propose plotting the Tafel slope value over small potential intervals (e.g., 20 mV) vs. the average current or potential, preferentially for different catalyst loadings, for all gas evolving and potentially diffusion limited processes.^[15]^ In this way, fundamental Tafel slope values can be separated from apparent Tafel slope values and a fairer and more rational activity comparison can be made between different catalyst/electrolyte combinations.

## Results and Discussion

Bubbles can exist as large bubbles on the catalyst and as micro or nanobubbles trapped inside the catalyst layer.^[16]^ The formation of oxygen bubbles can block part of the surface area of the porous catalyst, impede diffusion through the layer, affect the concentration (gradient) of reactant (i.e., hydroxide) and product at the interface and result in a supersaturation overpotential.^[17]^ The release of bubbles induces local mass transport. The stability of large bubbles on the surface of the catalyst should depend strongly on the rotation rate, while small microbubbles inside or even on the layer should not, or much less.^[17a]^

**Figure 1:**
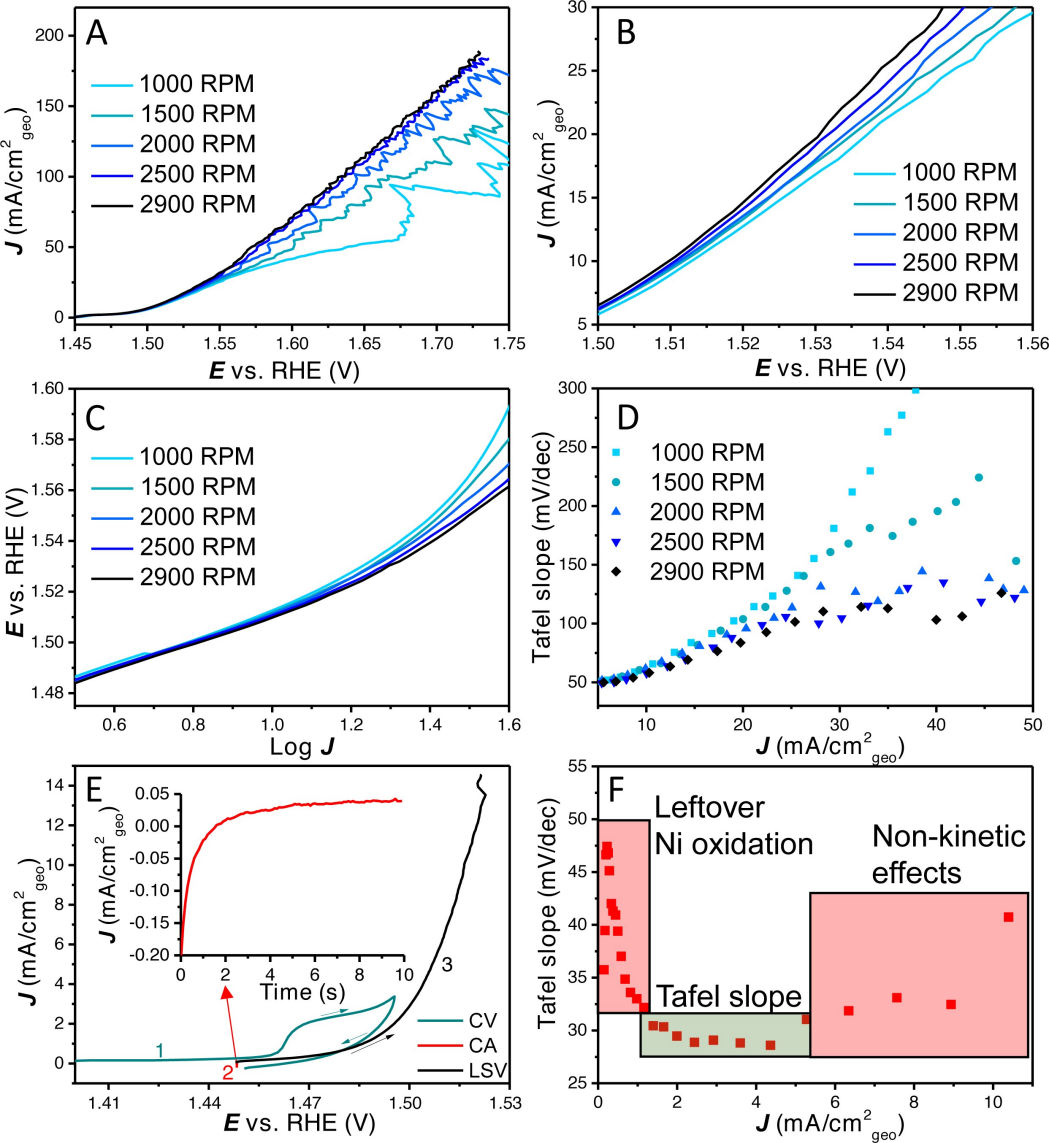
(A) 100 % *iR* corrected LSVs with rotation rates ranging from 1000 RPM to 2900 RPM, (B) zoomed in LSVs diverging at low overpotential with different rotation rates, (C) potential *E* vs. log *J* (Tafel plot), (D) Tafel slope vs. average current density (Tafel slope obtained over a 20 mV interval). (E) CV‐CA‐LSV procedure to remove most of the Ni oxidation peak, (F) the Tafel slope vs. average current density (Tafel slope obtained over a 5 mV interval, smaller interval is possible due to the lower noise level at low current density) showing Ni oxidation, initial Tafel slope value of ≈30 mV dec^−1^ and the onset of non‐kinetic effects. Continuous Tafel slope without Ni oxidation plots are shown in Figure S1.

In Figure 1, 100 % *iR* corrected linear sweep voltammograms are shown (5 mV s^−1^) of an electrodeposited catalyst layer prepared with a cathodic deposition current of 4 mA cm^−2^_Au_ for 5 s. As can be observed, there is a strong dependence of the current density on the rotation rate of the rotating disk electrode (RDE) from 1000 and 2900 RPM. At larger current densities the effect of bubbles can be observed by the current fluctuations (Figure 1A). Strikingly, the influence of rotation rate is already present at low current density (<10 mA cm^−2^), as shown in Figure 1B. The difference between 2500 and 2900 RPM is small and higher rotation rates would probably not suppress the current fluctuations much further.

Usually, Tafel slopes are determined by fitting (multiple) linear region(s) in a plot of the potential vs. the log of the current density (Figure 1C). In this plot, there is a similar slope at low current density, while with increasing current density very different slopes could be fitted for the different rotation rates. Clearly, this variance in Tafel slope values does not relate to a fundamental rate determining step anymore. Nevertheless, such slopes would still have quite high *R* ^*2*^ values, but they have limited meaningfulness in terms of mechanistic interpretation. Such possible misinterpretations as a result of fitting apparent linear regions in Tafel plots are further explored in Figure 7.

To separate potentially fundamental Tafel slopes from different non‐kinetic processes, we propose plotting the Tafel slope value over small intervals against the average current density. In Figure 1D, the Tafel slope values (obtained over a range of 20 mV) are plotted against the average current density. In this plot, it can be clearly observed that the Tafel slope converges to the same value at low current density while a continuous increase is observed at higher current density, depending on the rotation rate. No horizontal (single or double) Tafel slope region can be observed under these conditions (LSV with 5 mV s^−1^ in 0.1 M KOH). When the contribution of the Ni oxidation peak is mostly removed, a small horizontal Tafel slope region of ≈30 mV dec^−1^ can be found, as shown in Figure 1F. So, initially (<5 mA cm^−2^) the Tafel slope is ≈30 mV dec^−1^, after which there is a continuous increase in Tafel slope value due to non‐kinetic effects.

**Figure 2:**
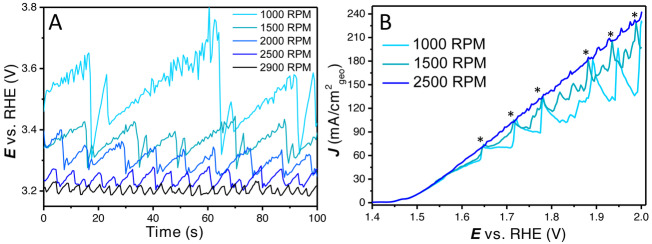
(A) Bubble formation and detachment at 35 mA (≈179 mA cm^−2^_geo_) from 1000 to 2900 RPM as observed by fluctuations in the uncompensated potential, (B) short manual rotation increases to show bubble release with 1000 RPM, 1500 RPM and 2500 RPM as stable rotation rates. Current spikes that hit the 2500 RPM LSV are induced by a brief increase in rotation rate (and are marked with an asterisk); smaller oscillations are caused by spontaneous bubble releases. LSVs are 85 % *iR* compensated to clearly show the vertical current spikes with bubble releases.

To illustrate the behaviour of large bubbles on this catalyst, the system was studied with chronopotentiometry measurements at 35 mA (or ≈179 mA cm^−2^_geo_) with different rotation rates. As expected, the uncompensated potential to achieve this current density is lower for increasing rotation rates, as shown in Figure 2A. It can be clearly observed that the bubble accumulation and detachment are dependent on the rotation rate. For low rotation rates the current oscillation amplitude is large and the frequency of bubble release is low. However, for higher rotation rates, the amplitude is lower and the frequency is higher.

In Figure 2B, (85 % *iR* compensated) LSVs are shown in which the rotation rate is increased manually for a short time. This short increase is used to remove the bubbles that are attached to the electrode. Next the rotation rate is returned to their previous stable value of 1000, 1500 and 2500 RPM. As can be observed, for 1000 and 1500 RPM this short increase in rotation rate coincides with a bubble release and therefore a sharp increase in current. After this interruption, the bubbles accumulate again, reducing the measured current density, before the next increase (as marked with an asterisk). In between the large amplitude current increases, there are also some small fluctuations/oscillations. These oscillations are caused by spontaneous smaller bubble releases. This experiment further confirms the effect external bubbles can have on activity at medium to high current densities.

Furthermore, the ohmic resistance of the system can increase with increasing potential due to bubbles on‐ and inside the layer, as well as bubbles altering the electrolyte path.^[17b]^ To study this effect, impedance was measured at different potentials. It was found that the ohmic resistance is not only dependent on potential, but it also depends on rotation rate, i.e., it is dependent on the presence of bubbles. The increase in ohmic resistance is minimal at high rotation rate compared to the large increase in ohmic resistance observed at low rotation rate (Figure S2 and S3). Often ohmic resistance is measured at open circuit potential (OCV) and this increase in ohmic resistance with potential will affect the Tafel slope analysis. As the above mentioned non‐kinetic effects influence each other, it is complicated to separately study them, and their effect on the Tafel slope analysis is mostly measured as a whole.

**Figure 3:**
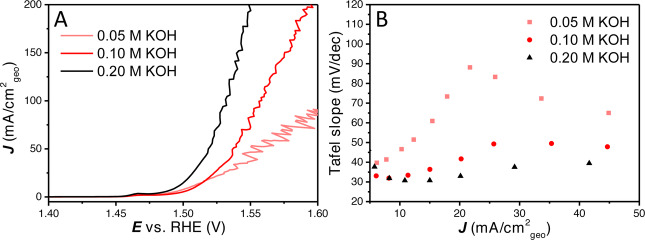
(A) 100 % *iR* compensated LSVs with different KOH concentrations of 0.05, 0.1 and 0.2 M. (B) The Tafel slope values with different hydroxide concentration vs. the average current density. Further CV, LSV and Tafel slope plots for the different KOH concentrations are given in Figure S4 and Figure S5.

Besides large and small bubbles on‐ and inside the catalyst layer and the related changes in ohmic resistance, OH^−^ gradients could exist in the porous LDH material at increasing current density.[^18^, ^19^] As internal diffusion is not altered with external convection, internal OH^−^ gradients will not depend on rotation rate.^[20]^ As has been described extensively, alkaline OER on Ni based materials is strongly pH dependent.^[21]^ To investigate the influence of the hydroxide concentration, LSV and Tafel slope analysis were performed in 0.05, 0.1 and 0.2 M KOH (Figure 3). It was found that the activity increases with increasing pH, as expected; at low current density the Tafel slope values seem to converge to the earlier observed ≈30 mV dec^−1^ for the different pH values. The increase in Tafel slope is faster for lower hydroxide concentration clearly showing its impact on the non‐intrinsic effects that influence the Tafel slope. Further CVs and Tafel slope plots at different pH values are shown in Figure S4 and Figure S5.

To further elucidate non‐intrinsic effects on “apparent” Tafel slope values, these effects were studied on layers of different loading. All different layers were produced by electrodeposition with varying times with a current of either 4 or 8 mA cm^−2^_Au_. After activation the active phase will be transformed to γ‐NiFe LDH,^[4]^ however the layers might have a slightly different morphology. To compare these layers in a fair manner they were all measured on the same day under the same conditions, because day‐to‐day variation can be a problem for the reproducibility, e.g., differences in bubble accumulation and release can be introduced by how far the rotator is inserted in solution (Figure S6).

**Figure 4:**
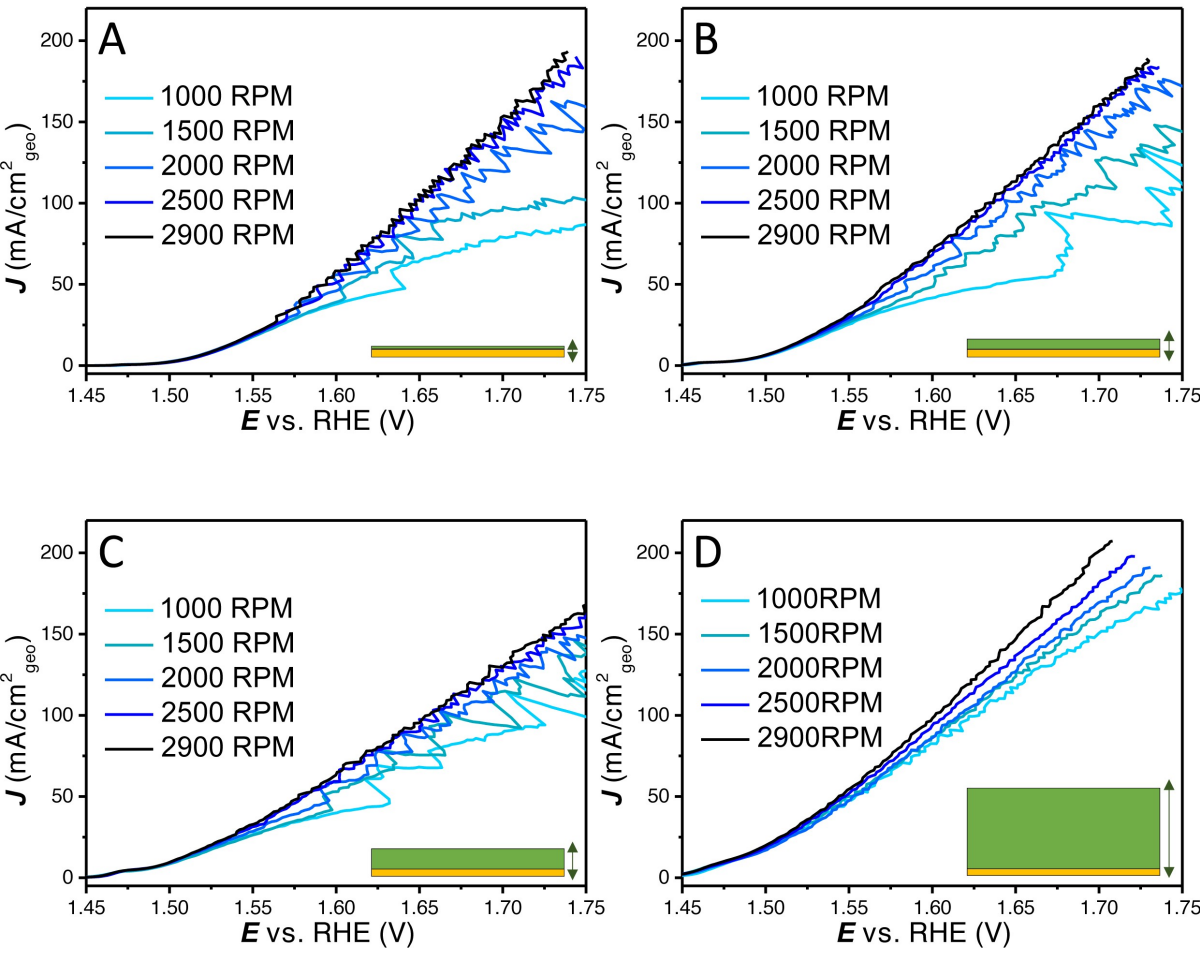
100 % *iR* compensated LSVs to show the rotation dependence (1000–2900 RPM) on the NiFeOOH layers with increasing loading obtained with different deposition charges (4.6, 20, 40 and 100 mC respectively from A to D).

**Figure 5:**
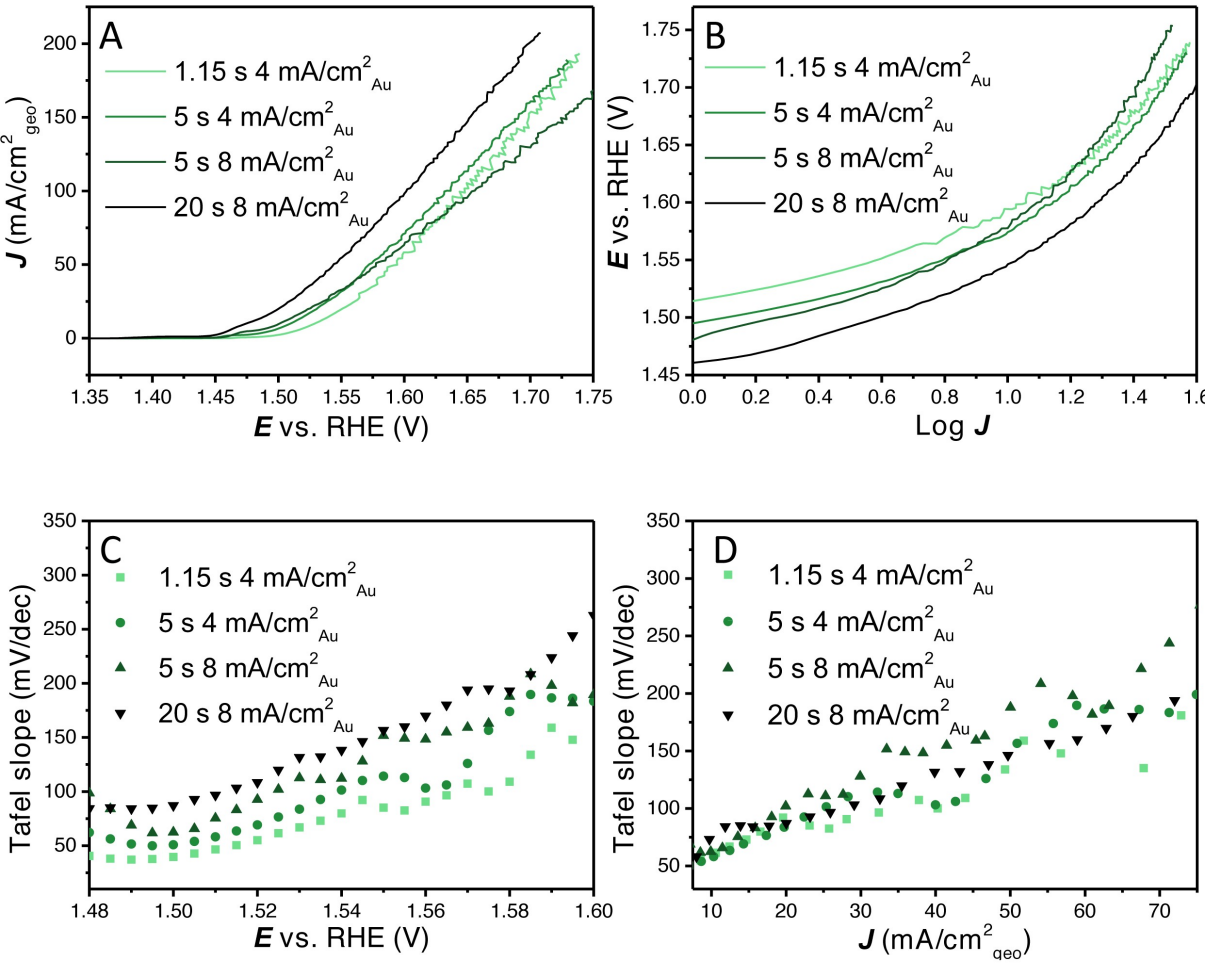
(A) 100 % *iR* compensated LSVs with different loadings at 2900 RPM, (B) potential *E* vs. log *J* (Tafel plot), (C) Tafel slope values vs. the potential (Tafel slope obtained over 20 mV intervals), (D) Tafel slopes values vs. the geometric current density (Tafel slope obtained over 20 mV intervals). Tafel slope values at low overpotential or current density can still be convoluted due to Ni oxidation current.

LSVs (100 % *iR* corrected) were recorded with different rotation rates on 4 distinct catalyst layers obtained with cathodic deposition charges of 4.6, 20, 40 and 100 mC (deposition current×time). As is shown in Figure 4, the rotation rate dependence of the geometrical current density is quite different for the different loadings. On the thinner layers, there is a relatively large difference in the different rotation rates (1000–2900 RPM). For the thicker layers, the rotational dependence becomes less signifcant. Besides that, the thinner catalyst layers (Figure 4A–B) show clear bubble formation and release, with large current oscillations at lower rotation and higher frequency of release at higher rotation rates. The thicker catalyst layers (Figure 4C–D) show only limited current oscillations associated with bubble release and only with small amplitudes. This could tentatively be explained by the differences in external to interal surface ratio, with thinner layers having less porosity and ECSA. It has been previously shown for H_2_ evolution that different arrays of CoS_2_ result in different bubble behaviour, whereas on large microwires bubbles are depinned, preventing large bubbles from forming on the top surface when compared to nanowires and flat surfaces.^[22]^ The comparison of the LSV response of different loadings at constant rotation rates is given in Figure 5, where different activities with respect to geometrical current density were obtained based on the rotation rate used.

The different layers were further analysed at the maximum rotation rate, i.e., 2900 RPM, as it is the least limited by the formation of large bubbles on its surface. In Figure 5A, the LSVs are shown, from which it can be observed that initially the activity increases with increasing loading. At higher current density the thinner layers are relatively less affected by non‐kinetic effects. The catalyst layer with the highest loading still remains the most active at the highest current density measured, however, that might change at even higher current density. In Figure 5B, we show how the Tafel slope values fitted to different regions in the traditional Tafel plot would strongly change with increasing current density for all layers.

Therefore, the Tafel slope values (obtained over a small potential region of 20 mV) are plotted against the average potential *E* and average current density *J* (Figure 5C and D). Strikingly, the plots of the Tafel slopes vs. the potential show that the thinner layers (which have a lower geometric current density) show lower Tafel slopes at similar overpotentials. It shows how the loading used, combined with the potential range scanned, has a profound effect on the obtained Tafel slope for a given catalyst material. However, when the Tafel slope is plotted vs. the geometric current density the difference between the layers is much smaller. At low current density, the Tafel slope value of all loadings converge to a similar Tafel slope value of 30–50 mV dec^−1^. However, no horizontal Tafel slope vs. *E* or *J* region can be observed under these conditions. A Tafel slope obtained from a linear region in Figure 5B would be some relatively arbitrary average of the different Tafel slope values in Figure 5C and D, depending on the chosen range. Further analysis of these layers with CV and integration of the nickel redox charge can be found in Figure S8.

The above results illustrate that a fundamental study of a catalyst layer in gas evolving reactions including the reporting of the Tafel slope requires thorough analysis of the system. Similar issues with non‐kinetic influences on Tafel slope analysis have been observed in fuel cell research, where thick catalyst layers exhibit mass transfer limitations.^[23]^ Different Tafel slope values have been found as a function of potential for IrOx/MnOx OER catalyst in chloride containing solution.^[15a]^ The kind of Tafel slope plot shown in Figure 1D and F, Figure 3B, Figure 5C and Figure 5D can help in establishing the kinetic meaningfulness of the Tafel slope analysis. Hence, Tafel slope values should be plotted against potential or current density to analyse if there is a continuous change in Tafel slope caused by non‐kinetic effects, e.g., bubble formation, ohmic resistance change and mass transport limitations, or if there is an intrinsic change in the catalyst activity, caused by e.g., a change in rate determining step or as a response to pseudocapacitive charging. True Tafel slope values can be found as horizontal regions in these Tafel slope plots or can be estimated by the converging value at the least limiting conditions.

**Figure 6:**
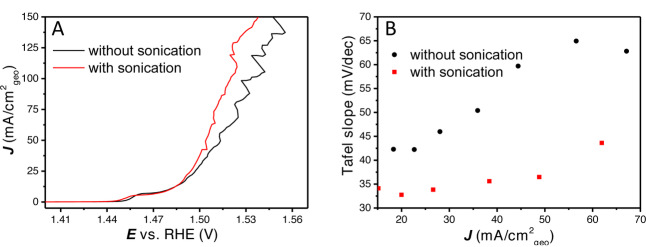
(A) 100 % *iR* compensated LSVs with and without sonication, (B) Tafel slope vs. the current density with and without sonication.

As has been described previously, non‐intrinsic effects (such as bubbles) have an important influence on measured activity and Tafel slope analysis. Another method to facilitate bubble detachments, including smaller gas bubbles, is sonication. In literature, sonication has been shown to improve the OER activity of the catalysts, as well as the apparent stability.[^16^, ^24^] It is essential to differentiate the effect of sonication from the effect of the increase in temperature. Therefore, measurements are performed at a higher scan rate of 20 mV s^−1^ in the same sonication bath and on the same catalyst. Increasing the scan rate reduces the number of peaks associated with bubble releases, but increases their amplitude (further details on scan rate dependence are given in Figure S9). Besides that, a higher scan rate results in an increase in the Ni oxidation current, so that the Tafel slope is convoluted by Ni oxidation current up to a higher current density (≈20 mA cm^−2^_geo_). Rotation rate was set at 2900 RPM, deposition conditions were 5 s at 4 mA cm^−2^_Au_, the bath temperature was measured to be 30 °C during these measurements (Figure 6). This increase in electrolyte temperature will increase the activity and lower the onset potential compared to room temperature (≈20 °C).

As can be observed in Figure 6A, the OER onset does not change by using sonication. The Tafel slope for both layers is initially close to 30 mV dec^−1^, after which it sharply increases with increasing current density without sonication, but stays significantly lower with sonication. However, at high current density, strong current oscillations caused by bubble release are still observed (Figure S10). To make sure the measured activity is not due to changes to the catalyst layer, a LSV was directly measured after the sonication measurement and an activity that is similar to the LSV without sonication in Figure 6 was observed (Figure S10). These experiments provide further evidence of how non‐kinetic effects on the surface cause this increase in Tafel slope. It shows that the removal of bubbles is essential for proper Tafel slope analysis, already at low to medium currents (<50 mA cm^−2^_geo_), which can be partially accomplished by sonication.

Interestingly, when plotting the Tafel slope over the full current range for the 2900 RPM with sonication, we still do not observe a large horizontal region in the Tafel slope plot, i.e., a constant Tafel slope value (Figure 6B). This shows that even when the non‐kinetic effects induced by bubbles are suppressed more effectively, there are still non‐kinetic processes occurring inside the catalyst layer that will influence the Tafel slope analysis. This could also be caused by OH^−^ mass transfer limitations inside the porous catalyst layer, which are not affect by sonication.

**Figure 7:**
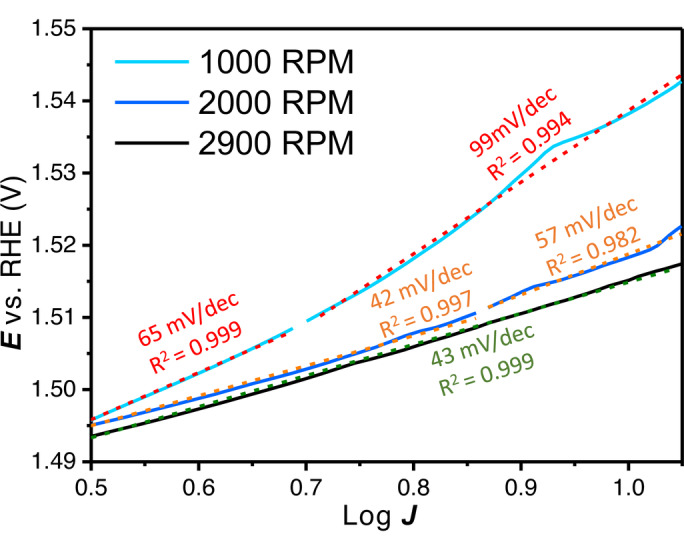
Tafel slope regions that could be defined between 0.5 and 1.05 log *J* (2–11.2 mA or 10–57.1 mA cm^−2^_geo_) with 1000 RPM, 2000 RPM and 2900 RPM on an electrodeposited catalyst (5 s at 8 mA cm^−2^_Au_).

To illustrate how different Tafel slopes can be assigned for the same catalysts, but under slightly different conditions, different ‘linear’ regions were fitted at different rotation rates. In Figure 7 Tafel slopes were fitted between 0.5 and 1.05 log *J* with rotation rates of 1000 RPM, 2000 RPM and 2900 RPM for an electrodeposited Ni_80_Fe_20_OOH layer for 5 s at 8 mA cm^−2^_Au_. In literature, rotation rates between 1500–2500 RPM are common when a RDE setup is used. By identifying (apparent) linear Tafel slope regions, very different kinetic data can be extracted for the same catalyst in the same electrolyte, with Tafel slopes still having quite high *R* ^*2*^ values. Therefore, the *R* ^*2*^ value is not a good metric for the meaningfulness of the obtained Tafel slope.

**Figure 8:**
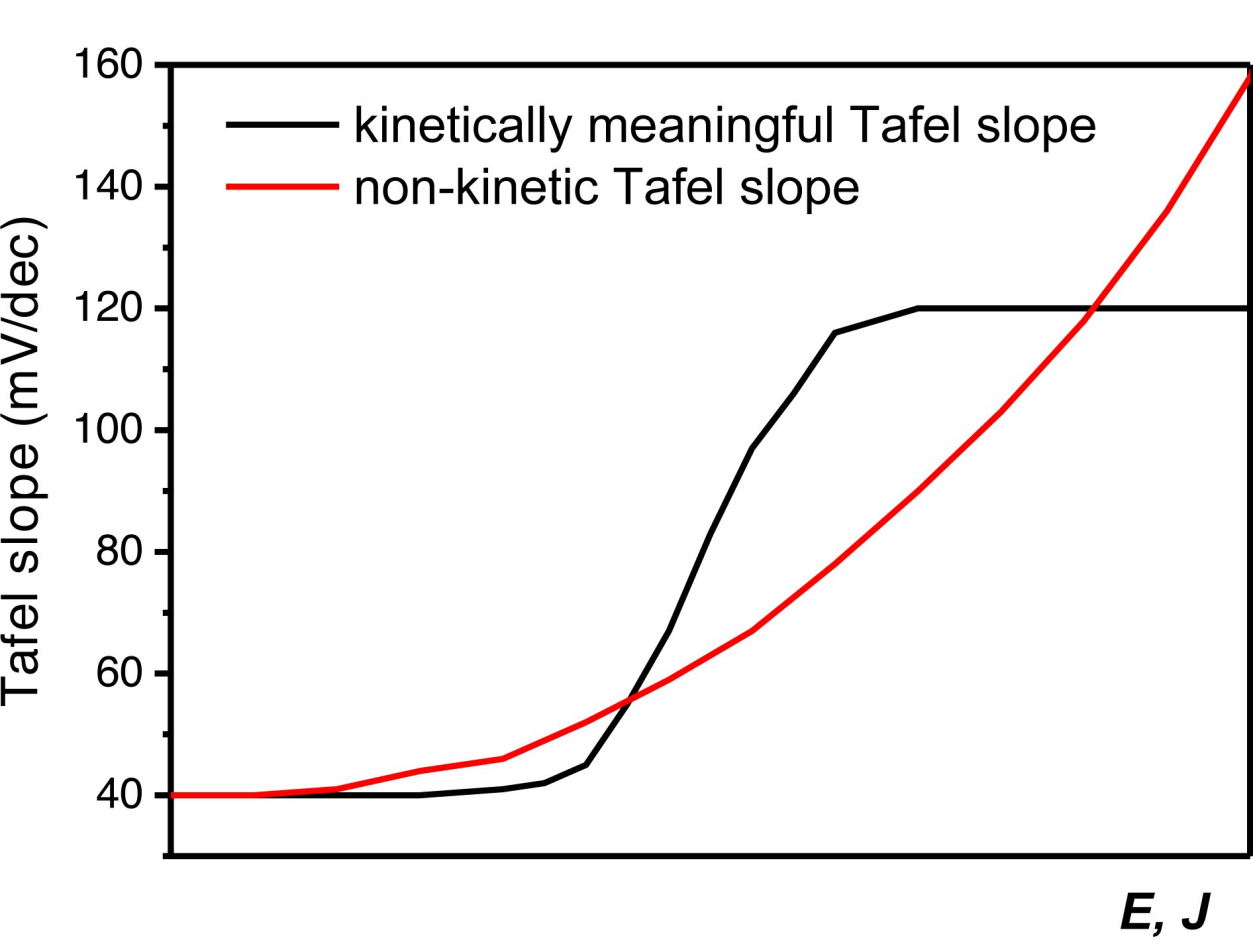
Schematic representation of how a kinetically meaningful Tafel slope (e.g., rate determining step change resulting in a Tafel slope of first 40 mV dec^−1^ and then 120 mV dec^−1^) can be differentiated from a non‐kinetic Tafel slope in a Tafel slope plot vs. potential or current density.

It is for this reason that we strongly advocate to make “Tafel slope plots” instead of Tafel plots, as in Figures 1D, 1F, 3B, 5C, 5D and 6B. If a (change in) Tafel slope is truly kinetically meaningful, this must show up as a horizontal region in such a plot, as illustrated in Figure 8.

In literature, low Tafel slopes in the range of 30–50 mV dec^−1^ have been reported for large surface area catalysts, as high surface area catalysts (if conductive and accessible) will already have a high geometric current density while the specific current density is still low and will be less limited by the discussed effects. For example, an active OER catalyst was reported with exfoliated layers, for which the Tafel slope was found to be 40 mV dec^−1^ over a large current and potential region.^[25]^ Furthermore, a hierarchically structured three dimensional NiFeOOH catalyst on Ni foam was reported with Tafel slope values of 33 and 28 mV dec^−1^ (in 0.1 M and 1 M KOH respectively).^[26]^ 34 and 44.1 mV dec^−1^ Tafel slopes were also reported for other NiFeOOH layers on Ni foam.^[27]^ Pulse deposited Ni(Fe)OOH layers were reported with a Tafel slope of 37±3 mV dec^−1^.^[28]^ Cu nanowires shelled with NiFeOOH have shown a Tafel slope of 27.8 mV dec^−1^.^[29]^ High surface area NiFe LDH on carbon nanotubes (CNT) were reported with a Tafel slope of 35 and 31 mV dec^−1^ in respectively 0.1 and 1 M KOH.^[30]^ Increasing the base concentration will reduce hydroxide mass transfer limitations and will thereby delay (but not remove) non‐kinetic effects. This will result in a lower Tafel slope as also shown in Figure 3 (for both Refs [26, 30]), but at higher current density the Tafel slope will still increase.

Besides catalyst synthesis methods to avoid bubble blocking and diffusional effects, sonication and magnetism have also been used to promote bubble removal and mass transport. The Tafel slope was found to be 41.7 mV dec^−1^ under the optimal magnetic field.^[31]^ Other techniques for advancing bubble detachments have been presented.^[32]^ Usually the Tafel slope values are reduced, but the onset is not changed significantly, showing the immense opportunity of bubble removal and effective mass transport for energy efficiency, as well as its importance for the reported activity metrics. For industrial purposes, suppressing bubble accumulation leads to a more efficient cell, as has been shown recently by the capillary fed water electrolysis cell.^[33]^

In summary, many Tafel slopes and activity assessments reported for the OER are “apparent”, as has been shown by the combination of the above discussed literature with the results presented in this paper. Different synthesis methods, bubble removal and mass transport increasing strategies significantly influence current responses for the OER, which shows the influence of non‐kinetic effects on Tafel slope analysis. It appears that the Tafel slope, when not limited by non‐kinetic effects, converges to 30 mV dec^−1^ for Ni_20_Fe_80_OOH in 0.05–0.2 M base and then continuously increases with increasing current. We recommend that claims of improved intrinsic activity of nano‐porous electrocatalysts should be accompanied by a Tafel slope plot that indicates the absence of non‐kinetic effects.

## Conclusion

We have shown that the large variety of reported Tafel slopes on the same catalyst material are likely caused by (loading dependent) bubble formation within and on the catalyst layer, resulting changes in ohmic resistance and (internal) OH^−^ gradients. We demonstrated that initially (<5 mA cm^−2^_geo_) the Tafel slope for Ni_80_Fe_20_OOH in 0.05–0.2 M KOH is ≈30 mV dec^−1^, while at increasing current densities there is a continuous increase in Tafel slope value that depends on the hydroxide concentration, rotation rate, loading and sonication. We show that such non‐kinetic effects can already play a role at low current density (<10 mA cm^−2^_geo_), something that may come as a surprise for a reaction that would normally not be considered as being hindered by mass‐transport limitations. No second Tafel slope region is observed and there is no indication for a change in rate determining step. As best practise, we propose that Tafel slopes should be plotted in Tafel slope plots. In such a plot, the Tafel slope is determined over small potential regions and plotted vs. the (average) current or potential, preferably for different loadings. From a Tafel slope plot, kinetically meaningful Tafel slope values can be determined from a horizontal Tafel slope region or by a region converging to a meaningful value under condition where mass transfer effects and bubble formation can effectively be ruled out. This approach is essential for any gas evolving or potentially mass transport limited reaction, especially when using less well‐defined nano‐porous catalyst layers instead of flat surfaces.

## Conflict of interest

The authors declare no conflict of interest.

1

## Supporting information

As a service to our authors and readers, this journal provides supporting information supplied by the authors. Such materials are peer reviewed and may be re‐organized for online delivery, but are not copy‐edited or typeset. Technical support issues arising from supporting information (other than missing files) should be addressed to the authors.

Supporting Information

## Data Availability

The data that support the findings of this study are available from the corresponding author upon reasonable request.

## References

[1] J. Kibsgaard , I. Chorkendorff , Nat. Energy 2019, 4, 430–433.

[2] L. Trotochaud , S. L. Young , J. K. Ranney , S. W. Boettcher , J. Am. Chem. Soc. 2014, 136, 6744–6753. 24779732 10.1021/ja502379c

[3] M. W. Louie , A. T. Bell , J. Am. Chem. Soc. 2013, 135, 12329–12337. 23859025 10.1021/ja405351s

[4] F. Dionigi , Z. Zeng , I. Sinev , T. Merzdorf , S. Deshpande , M. B. Lopez , S. Kunze , I. Zegkinoglou , H. Sarodnik , D. Fan , A. Bergmann , J. Drnec , J. Ferreira de Araujo , M. Gliech , D. Teschner , J. Zhu , W. Li , J. Greeley , B. Roldan Cuenya , P. Strasser , Nat. Commun. 2020, 11, 2522. 32433529 10.1038/s41467-020-16237-1 PMC7239861

[5a] S. Watzele , P. Hauenstein , Y. Liang , S. Xue , J. Fichtner , B. Garlyyev , D. Scieszka , F. Claudel , F. Maillard , A. S. Bandarenka , ACS Catal. 2019, 9, 9222–9230;

[5b] S. Watzele , A. S. Bandarenka , Electroanalysis 2016, 28, 2394–2399;

[5c] E. Cossar , M. S. E. Houache , Z. Zhang , E. A. Baranova , J. Electroanal. Chem. 2020, 870, 114246.

[6] C. C. L. McCrory , S. Jung , J. C. Peters , T. F. Jaramillo , J. Am. Chem. Soc. 2013, 135, 16977–16987. 24171402 10.1021/ja407115p

[7a] N. Govindarajan , G. Kastlunger , H. H. Heenen , K. Chan , Chem. Sci. 2022, 13, 14–26; 10.1039/d1sc04775b PMC8694373 35059146

[7b] A. R. Akbashev , ACS Catal. 2022, 12, 4296–4301.

[8a] T. Shinagawa , A. T. Garcia-Esparza , K. Takanabe , Sci. Rep. 2015, 5, 13801; 26348156 10.1038/srep13801 PMC4642571

[8b] A. M. Limaye , J. S. Zeng , A. P. Willard , K. Manthiram , Nat. Commun. 2021, 12, 703. 33514735 10.1038/s41467-021-20924-y PMC7846806

[9] G. Kear , F. C. Walsh , Corros. Mater. 2005, 30, 51–55.

[10] S. Anantharaj , S. R. Ede , K. Karthick , S. S. Sankar , K. Sangeetha , P. E. Karthik , S. Kundu , Energy Environ. Sci. 2018, 11, 744–771.

[11a] N. T. Suen , S. F. Hung , Q. Quan , N. Zhang , Y. J. Xu , H. M. Chen , Chem. Soc. Rev. 2017, 46, 337–365; 28083578 10.1039/c6cs00328a

[11b] X. Gu , Z. Liu , M. Li , J. Tian , L. Feng , Appl. Catal. B 2021, 297, 120462.

[12] H. N. Nong , L. J. Falling , A. Bergmann , M. Klingenhof , H. P. Tran , C. Spöri , R. Mom , J. Timoshenko , G. Zichittella , A. Knop-Gericke , S. Piccinin , J. Pérez-Ramírez , B. Roldan Cuenya , R. Schlögl , P. Strasser , D. Teschner , T. E. Jones , Nature 2020, 587, 408–413. 33208960 10.1038/s41586-020-2908-2

[13a] F. Dionigi , P. Strasser , Adv. Energy Mater. 2016, 6, 1600621;

[13b] Y. Liu , D. Zhou , T. Deng , G. He , A. Chen , X. Sun , Y. Yang , P. Miao , ChemSusChem 2021, 14, 5359–5383; 34704377 10.1002/cssc.202101898

[13c] M. Gong , H. Dai , Nano Res. 2015, 8, 23–39;

[13d] X. Deng , J. Huang , H. Wan , F. Chen , Y. Lin , X. Xu , R. Ma , T. Sasaki , J. Energy Chem. 2019, 32, 93–104;

[13e] M. Yu , E. Budiyanto , H. Tüysüz , Angew. Chem. Int. Ed. 2022, 61, e202103824; 10.1002/anie.202103824 PMC9291824 34138511

[13f] S. K. Pramanik , F. B. Suja , S. Zain , B. K. Pramanik , Mater. Today Energy 2022, 101036.

[14] P. Chakthranont , J. Kibsgaard , A. Gallo , J. Park , M. Mitani , D. Sokaras , T. Kroll , R. Sinclair , M. B. Mogensen , T. F. Jaramillo , ACS Catal. 2017, 7, 5399–5409.

[15a] J. G. Vos , T. A. Wezendonk , A. W. Jeremiasse , M. T. M. Koper , J. Am. Chem. Soc. 2018, 140, 10270–10281; 30024752 10.1021/jacs.8b05382 PMC6099550

[15b] J. G. Vos , A. Venugopal , W. A. Smith , M. T. M. Koper , J. Catal. 2020, 389, 99–110;

[15c] J. G. Vos , A. Venugopal , W. A. Smith , M. T. M. Koper , J. Electrochem. Soc. 2020, 167, 046505.

[16] H. A. El-Sayed , A. Weiß , L. F. Olbrich , G. P. Putro , H. A. Gasteiger , J. Electrochem. Soc. 2019, 166, F458.

[17a] A. R. Zeradjanin , ChemSusChem 2018, 11, 1278–1284; 29436179 10.1002/cssc.201702287

[17b] X. Zhao , H. Ren , L. Luo , Langmuir 2019, 35, 5392–5408. 30888828 10.1021/acs.langmuir.9b00119

[18] R. D. L. Smith , R. S. Sherbo , K. E. Dettelbach , C. P. Berlinguette , Chem. Mater. 2016, 28, 5635–5642.

[19] R. Chen , S. Hung , D. Zhou , J. Gao , C. Yang , H. Tao , H. B. Yang , L. Zhang , L. Zhang , Q. Xiong , H. M. Chen , B. Liu , Adv. Mater. 2019, 31, 1903909. 10.1002/adma.201903909 31461181

[20] C. T.-C. Wan , K. V. Greco , A. Alazmi , R. M. Darling , Y.-M. Chiang , F. R. Brushett , J. Electrochem. Soc. 2021, 168, 123503.

[21a] L. Giordano , B. Han , M. Risch , W. T. Hong , R. R. Rao , K. A. Stoerzinger , Y. Shao-Horn , Catal. Today 2016, 262, 2–10;

[21b] O. Diaz-Morales , D. Ferrus-Suspedra , M. T. M. Koper , Chem. Sci. 2016, 7, 2639–2645; 28660036 10.1039/c5sc04486c PMC5477031

[21c] C. Yang , M. Batuk , Q. Jacquet , G. Rousse , W. Yin , L. Zhang , J. Hadermann , A. M. Abakumov , G. Cibin , A. Chadwick , J. Tarascon , A. Grimaud , ACS Energy Lett. 2018, 3, 2884–2890.

[22] M. S. Faber , R. Dziedzic , M. A. Lukowski , N. S. Kaiser , Q. Ding , S. Jin , J. Am. Chem. Soc. 2014, 136, 10053–10061. 24901378 10.1021/ja504099w

[23a] D. W. Banham , J. N. Soderberg , V. I. Birss , J. Phys. Chem. C. 2009, 113, 10103–10111 DOI: 10.1021/jp809987g;

[23b] M. Darab , A. O. Barnett , G. Lindbergh , M. S. Thomassen , S. Sunde , Electrochim. Acta 2017, 232, 505–516.

[24] A. Hartig-Weiss , M. F. Tovini , H. A. Gasteiger , H. A. El-Sayed , ACS Appl. Energy Mater. 2020, 3, 10323–10327.

[25] F. Song , X. Hu , Nat. Commun. 2014, 5, 4477. 25030209 10.1038/ncomms5477

[26] X. Lu , C. Zhao , Nat. Commun. 2015, 6, 6616. 25776015 10.1038/ncomms7616 PMC4382694

[27a] W. Li , F. Li , H. Yang , X. Wu , P. Zhang , Y. Shan , L. Sun , Nat. Commun. 2019, 10, 5074; 31699987 10.1038/s41467-019-13052-1 PMC6838099

[27b] J. Jin , J. Xia , X. Qian , T. Wu , H. Ling , A. Hu , M. Li , T. Hang , Electrochim. Acta 2019, 299, 567–574.

[28] A. S. Batchellor , S. W. Boettcher , ACS Catal. 2015, 5, 6680–6689.

[29] L. Yu , H. Zhou , J. Sun , F. Qin , F. Yu , J. Bao , Y. Yu , S. Chen , Z. Ren , Energy Environ. Sci. 2017, 10, 1820–1827.

[30] M. Gong , Y. Li , H. Wang , Y. Liang , J. Z. Wu , J. Zhou , J. Wang , T. Regier , F. Wei , H. Dai , J. Am. Chem. Soc. 2013, 135, 8452–8455. 23701670 10.1021/ja4027715

[31] X. Qin , J. Teng , W. Guo , L. Wang , S. Xiao , Q. Xu , Y. Min , J. Fan , Catal. Lett. 2022.DOI : 10.1007/s10562-022-04032-0

[32] G. B. Darband , M. Aliofkhazraei , S. Shanmugam , Renewable Sustainable Energy Rev. 2019, 114, 109300.

[33] A. Hodges , A. L. Hoang , G. Tsekouras , K. Wagner , C. Lee , G. F. Swieger , G. G. Wallace , Nat. Commun. 2022, 13, 1304. 35292657 10.1038/s41467-022-28953-x PMC8924184

