# Supplementary material for: Non‐Kinetic Effects Convolute Activity and Tafel Analysis for the Alkaline Oxygen Evolution Reaction on NiFeOOH Electrocatalysts
Source: Angew Chem Int Ed Engl. 2023 Jan 10;62(7):e202216477. doi: 10.1002/anie.202216477 (PMC10108042; doi:10.1002/anie.202216477)
Supplement: Supplementary file 1 — Supporting Information [file ANIE-62-0-s001.pdf]

Supporting information

Table of contents

Experimental Section 2

Low current continuous Tafel slope 4

Impedance at different potentials 5

Different KOH concentrations 7

Experimental issues for reproducibility 8

LSVs of different layer thicknesses at constant rotation rates from 1000 to 2500 RPM. 9

CVs of NiFeOOH catalysts of different loading normalised to geometric surface area and charge, Ni redox charge to deposition charge. 10

LSV at different scan rates 11

Full LSV for sonication 12

Experimental Section

Preparation of the working electrode

The RDE setup (MSR Rotator, Pine Research) was used, with a Teflon rotator tip with a gold insert (Pine Research, 5 mm OD; 4mm thick; 0.196 cm^2). First, the gold disk was polished on a microcloth (Buehler) with metaDi polishing solution (Buehler) of respectively 3, 1 and 0.25 µm for ~2 minutes each. Afterwards, the tip was sonicated for approximately 10 minutes to remove residual diamond particles. The surface area of the gold disk was determined in an 0.1 M H_2SO_4 (Merck, suprapur, 96%) solution in a three electrode cell with a platinum counter and a reversible hydrogen electrode (RHE) homemade from a Luggin capillary with platinum wire (50 µm diameter, Goodfellow, 99.99%) and H_2 gas bubbled through the solution. 5 CV scans between 0.05 and 1.75V with 50 mV/s were taken and when stable, the surface area was determined by integrating the reduction peak, thereby obtaining the reduction charge. The reduction charge was divided by 0.39 mC/cm^2,[1] to obtain the real surface area value. The real surface area was usually between 0.35-0.40 cm^2, compared to 0.196 cm^2 geometrical surface area.

The deposition solution was 80 mM $Ni(NO3)_2.6H_2O$ (Sigma-Aldrich, 99.999% trace metal basis) and miliQ water (> 18.2 MΩ.cm), which is first purged with argon for at least 20 minutes. To this solution $Fe(SO_4).7H_2O$ (Sigma-Aldrich, ACS reagent, ≥ 99%) salt was added, resulting in 20 mM Fe:80 mM Ni solution in miliQ water. Deposition was performed in a two electrode cell with a platinum counter electrode, the working electrode was a rotating disk electrode (Au disk), deposition was performed under rotation rate of 400 RPM. An 8 or 4 mA/cm^2_{Au} current to the real gold surface area was applied for different time periods to deposit layers of different loadings.

Oxygen Evolution Reaction (OER)

For the OER measurements, a three electrode setup was used, with the deposited NiFeOOH RDE as working electrode, a high surface area gold wire counter electrode (0.8 mm thick, Mateck, 99.9%), a Hydroflex® RHE reference electrode (Gaskatel), which was connected to a 10 mF capacitor and a small sacrificial gold wire inside the solution to reduce noise. A 0.1M KOH solution was used as electrolyte in a plastic cell (Nalgene®) as alkaline solution leaches silicates from the glass, which can affect the electrochemistry.[2] First an activation procedure was performed of 30-50 scans at 50 mV/s, until the charge and OER current are fully stabilized. Impedance measurements at OCV, from 100 kHz to 1 Hz were performed to determine the ohmic resistance, which is defined as the intersect of the Nyquist plot with the real axis at high frequency.[3]

A set of linear sweep voltammograms (LSV) was performed between 1.2 and 2 V at different rotation speeds in the order of 1000 RPM – 2500 RPM – 1500 RPM – 2900 RPM – 2000 RPM, to avoid catalyst deactivation influencing the interpretation. The scan rate was 5 mV/s and the ohmic resistance was compensated for 85% by the EC-lab software, the remaining 15% was compensated manually. In between subsequent LSV measurements, chronoamperometry was performed at 1.2 V to stabilize the system and to remove all remaining bubbles. Ohmic resistance was measured at OCV again after the 1500 RPM LSV and after the last LSV scan to make sure that there was no shift in ohmic resistance, since small shifts in ohmic resistance have a large effect on the reported current at medium to high current densities and therefore also on the reported Tafel slope.

To remove the contribution of the Ni oxidation peak from the Tafel slope analysis, first a CV was taken from 1.2 to 1.525 V and back to 1.448V at 10 mV/s, then a CA was performed at 1.448 V for 10s, finally a LSV was conducted from 1.448 to 1.65 V at 5 mV/s (or at a scan rate as specified in the SI), the rotation rate was always 2900 RPM. After a 30s CA at 1.2 V to remove left over bubbles, the impedance was measured at OCV (similar as described earlier). The LSV was not *iR*-compensated in-situ, afterwards the potential was manually compensated for 100%.

To determine the contribution of OH^- gradients, LSVs with 5 mV/s and 2900 RPM, similar to other LSV measurements, were taken from 1.2 to 1.8 V for 0.2 M KOH, to 2.0 V for 0.1 M KOH and to 2.2 V for 0.05 M KOH, as the ohmic resistance changes between these different electrolytes. 85% of the *iR* compensation was performed in-situ by the EC-lab software, the remaining 15% was compensated manually afterwards. The KOH was prepared from the same 1 M stock solutions of Fe-free KOH.

Cyclic voltammetry (CV) was performed from 1.20 to 1.65 V (85% *iR* compensation, 15% is manually compensated afterwards) at 10 mV/s in the same setup. The redox charge was calculated by determining the reversible charge in the integrated current CV, which belong to redox charge, the non-reversible charge is due to oxygen evolution or possibly catalyst dissolution.[4]

Impedance at different potentials was measured at 7 equally spaced (uncompensated) potentials between 1.35 V - 2.5 V, chronoamperometry (CA) at relevant potential for 30 seconds prior to impedance to stabilise the layer. Impedance was recorded from 50 000 kHz to 1 Hz with a 10 mV amplitude to determine possible changes in ohmic resistance with potential (and current).

Sonication measurements

For the sonication measurements, the OER cell (as described before) was fixed in a sonication bath (Bandelin Sonorex, 35 kHz, 60 W). As sonication also induces a temperature effect the two measurements that are compared were both performed in the same sonication bath in short succession. The rotation rate during all these experiments was 2900 RPM. The first LSV was taken with the sonication off. The next LSV was measured with the sonication turned on. Afterwards a 3rd LSV was performed without sonication to make sure that there were no permanent changes in the system. In between the scans there was a short CA to remove left over bubbles and the ohmic resistance (impedance) was measured prior to every LSV (with sonication on for the sonication LSV). To shorten the period in which the bath was heated, all scans were performed at 20 mV/s. The bath temperature for the sonication experiment was monitored and was ~30°C for this measurement. The LSVs were fully compensated for the ohmic resistance value obtained prior to the scan (85% *iR* compensation during the scan).

Tafel slope analysis

The Tafel slope is empirically described as follows:[5]

$$\eta = a + b * \log(j) \qquad (1)$$

with η the overpotential in mV, j the current density in mA/cm^2, a the exchange current density and b the Tafel slope in mV/dec.

Insight about the rate-determining steps in a mechanism can be derived from Tafel slopes, as has been done for OER (and HER, HOR & ORR).[5] However, to determine an accurate Tafel slope value the reaction should be fully in the charge transfer limited regime, i.e., not limited by mass transfer, internal resistances or surface blockage.[6] Tafel slopes must always be obtained under 100% *iR* compensation.[7] Here, we determined the Tafel slope over small potential ranges (5 or 20 mV), which were then plotted as a function of the average current or vs. the potential. In this way, the evolution of the Tafel slope value can be followed at different currents or potentials, while not depending on arbitrarily chosen linear regions. Processes that interfere with the purely kinetic interpretation of the Tafel slope include internal and external bubble formation, change in ohmic resistance, and mass transport limitation; for alkaline OER such mass transport limitations are related to OH^- concentration gradients.

Low current continuous Tafel slope

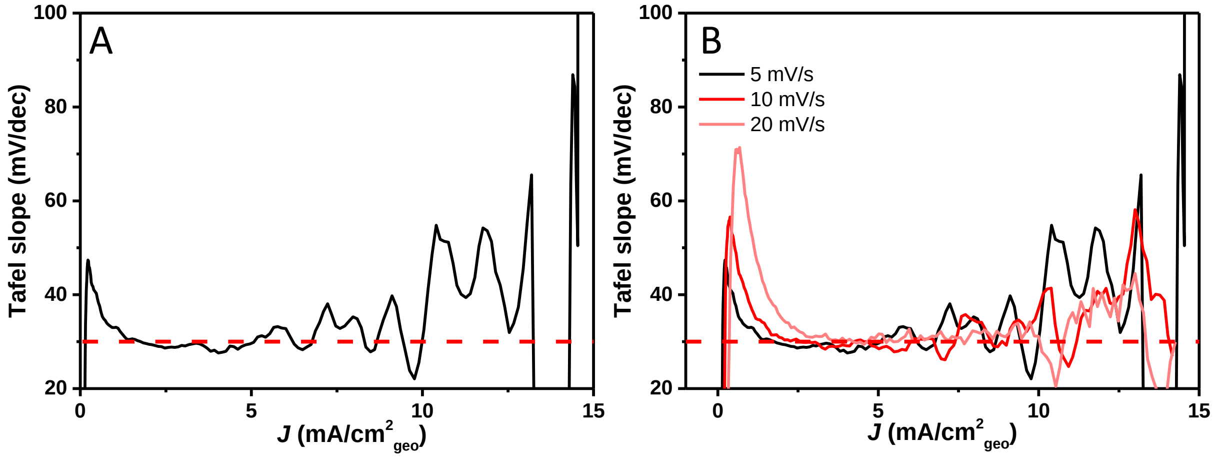

Figure S1. Continuous Tafel slope determined between 0 - 15 mA/cm^2 with (A) 5 mV/s and (B) with 5, 10 and 20 mV/s showing a similar ~30 mV/dec horizontal Tafel slope region until approximately 5 mA/cm^2, after which the Tafel slope value starts to increase and the noise starts to increase as well. That initially there is an influence of the (remaining) Ni oxidation peak can be observed by the scan rate dependence, the horizontal Tafel slope region does not change with scan rate, as expected. However, there seems to be less increase in Tafel slope due to non-kinetic effects with higher scan rates.

Impedance at different potentials

Ohmic resistance can be influenced by internal and external bubble formation.[8] The lower frequency region is so noisy that it is not shown here, as the catalyst layer is constantly changing due to bubble formation the measurement is not stable over the time range recorded at low frequency. In Figure S2, the rotation-dependent increase of ohmic resistance at increasing potential (1.35 - 2.5 V vs. RHE uncompensated) is shown for 1000, 2000 and 2900 RPM. The ohmic resistance, which is given as the intersect of the Nyquist plot with the real axis at high frequency,[9] is similar at 1.35 V after which the ohmic resistance starts to increase at higher potential. The increase is quite large and happen in multiple steps for 1000 RPM, whereas for 2900 RPM this increase is relatively small and the maximum ohmic resistance is already reached at the 3rd potential step, but it is still not fully negligible. The ohmic resistance increase at 2000 RPM is intermediate. The impedance data shows how in-situ changes in ohmic resistance can also convolute Tafel slope determination, as all ohmic resistances should be compensated for fundamental insights to be obtained. The activity becomes very similar when compensated for the ohmic resistance value found at each potential step (Figure S3)**.** However, the Tafel slope value is not constant over this range, so the ohmic resistance is definitely not the only limiting parameter for the Tafel slope determination.

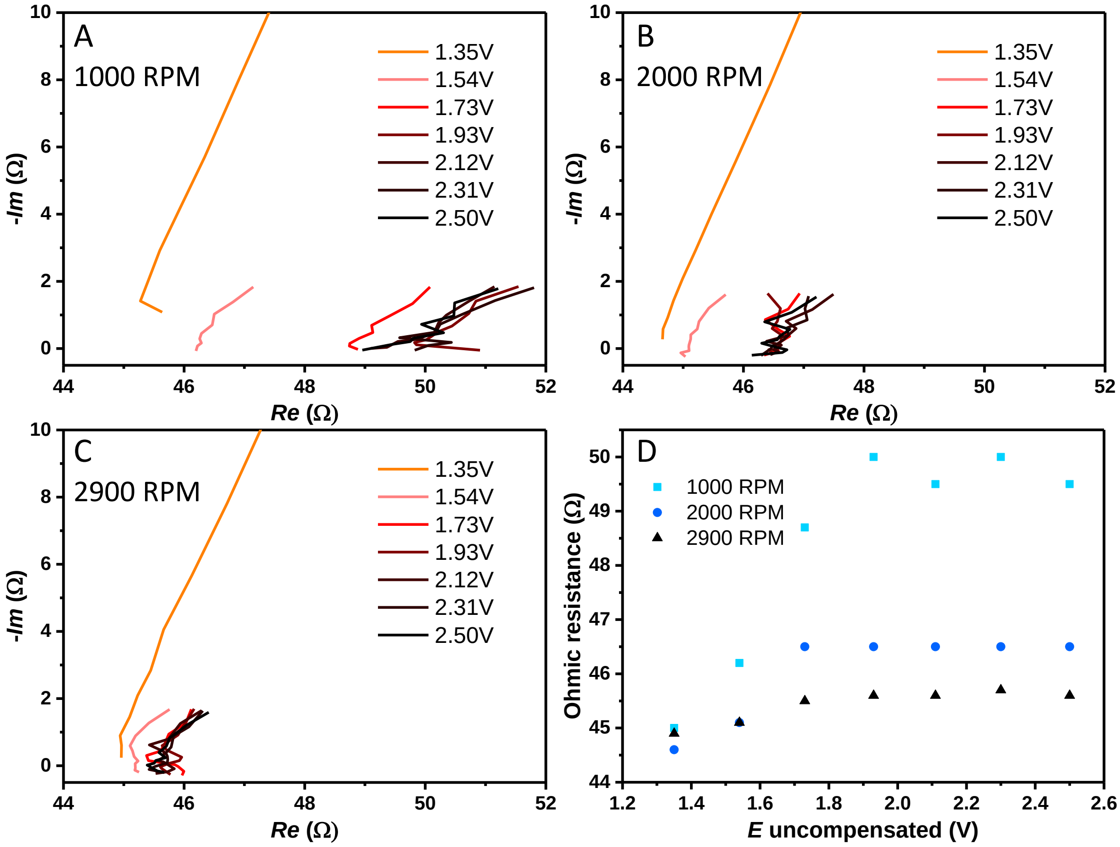

Figure S2. Nyquist plots of the impedance measurements in the 50 000 to 1000 Hz high frequency region at different rotation rate with (A) 1000 RPM, (B) 2000 RPM (C) 2900 RPM and (D) the obtained resistances vs. the uncompensated potential for all rotation rates. The ohmic resistance increase is rotation rate dependent and also shows that the layer resistance increases due to bubble formation. The measurements are taken by 7 equally spaced cycles between 1.35 V to 2.5 V (no *iR* compensation). Catalyst layer deposition conditions were small small layer): 4 mA/cm^2, 1 s.

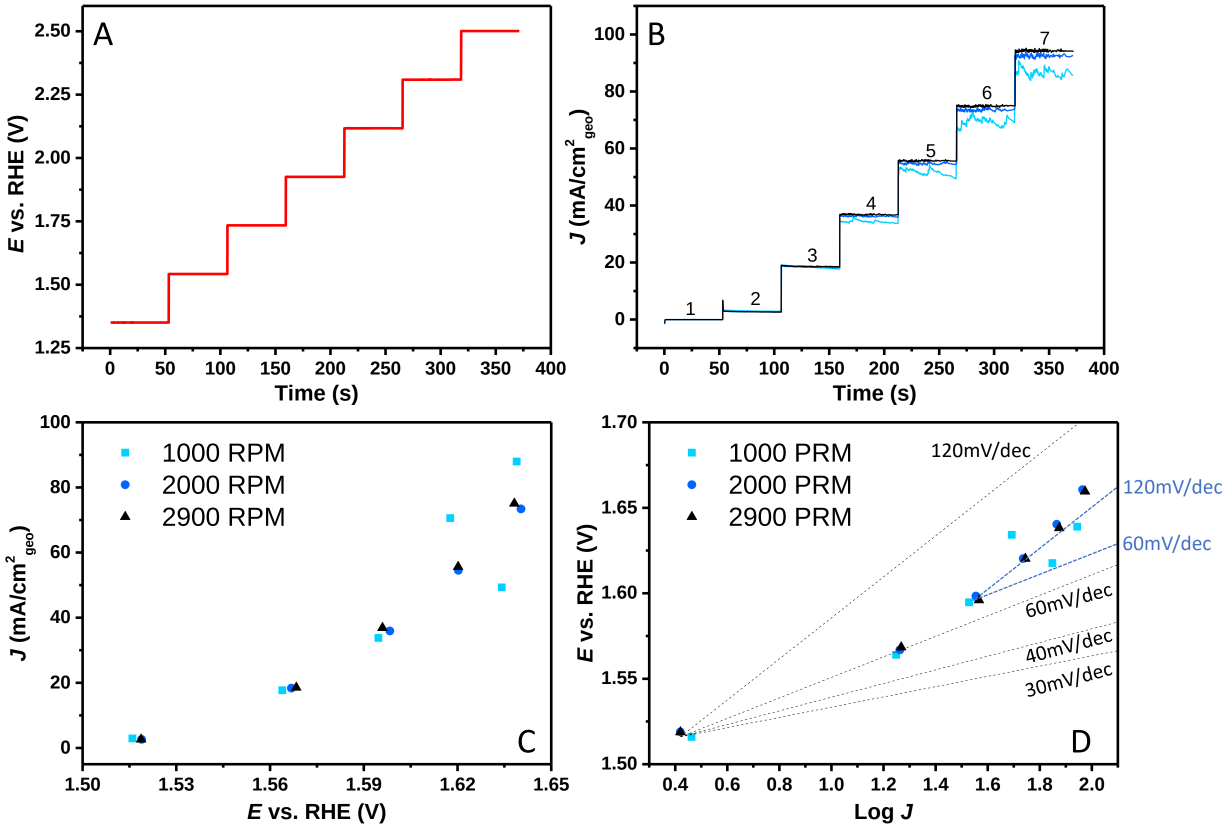

Figure S3. (A) potential steps vs. time, (B) current density corresponding to the potential steps, (C) *J* vs. *E* and (D) *E* vs. log *J* plot of fully compensated potential pulses with the actual ohmic resistance measured at that potential, lines to guide the eye are given for the cardinal Tafel slope values anchored at the 1st (30, 40, 60 and 120 mV/dec) and 3rd (60 and 120 mV/dec) datapoint. There does not seem to be a cardinal Tafel slope value in either region (Figure S3D).

Different KOH concentrations

To investigate the effect of the hydroxide concentration, measurements were performed in 0.05, 0.1 and 0.2 M KOH.

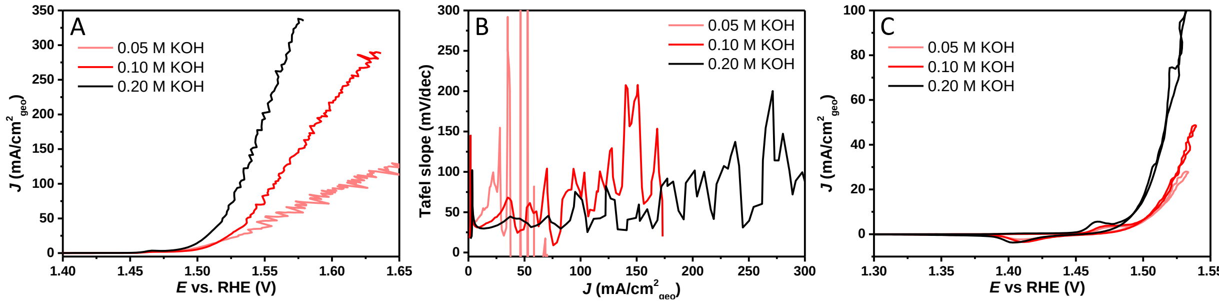

Figure S4. (A) full LSV at different KOH concentration, (B) continuous Tafel slope determination from the full LSV (C) CV of 5 s 4 mA/cm$^2_{Au}$ with different KOH concentration.

To obtain additional insight into the Tafel slope at low current density, the CV-CA-LSV procedure (similar to Figure 1E) was used to remove the majority of the Ni oxidation peak. The final LSV here was performed to 1.75 V with a scan rate of 5 mV/s (uncompensated). Here it can be observed that the Tafel slopes for all tested hydroxide concentrations seem to start at ~30 mV/dec. The onset and magnitude of non-kinetic contributions to the Tafel slope depends on the base concentration.

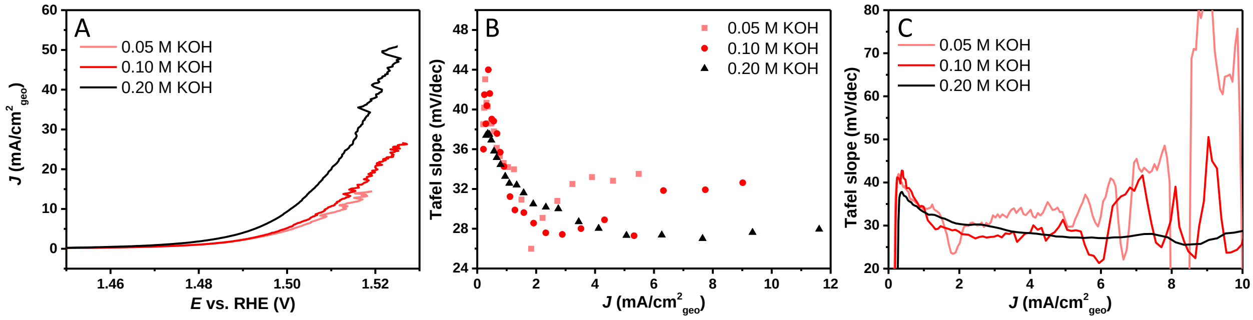

Figure S5. (A) LSV with the majority of the Ni oxidation contribution removed, (B) Tafel slope vs. current density for the LSVs in A (Tafel slopes determined over 5 mV intervals), (C) continuous Tafel slope determination.

Experimental issues for reproducibility

Besides a large dependency on the rotation rate, day-to-day changes, such as the rotator used and how far the rotator shaft is inserted into the solution also affect the results. To show that, in Figure S6, two sets of LSVs (85% *iR* compensated) with different rotation rates are given with the rotator less or more inserted (experiments 1500/2500/2900 RPM-2 are more inserted). This has a large influence on the bubble formation and release. Therefore, experiments here that are directly compared are always done on the same rotator, in the same height settings and in the same cell.

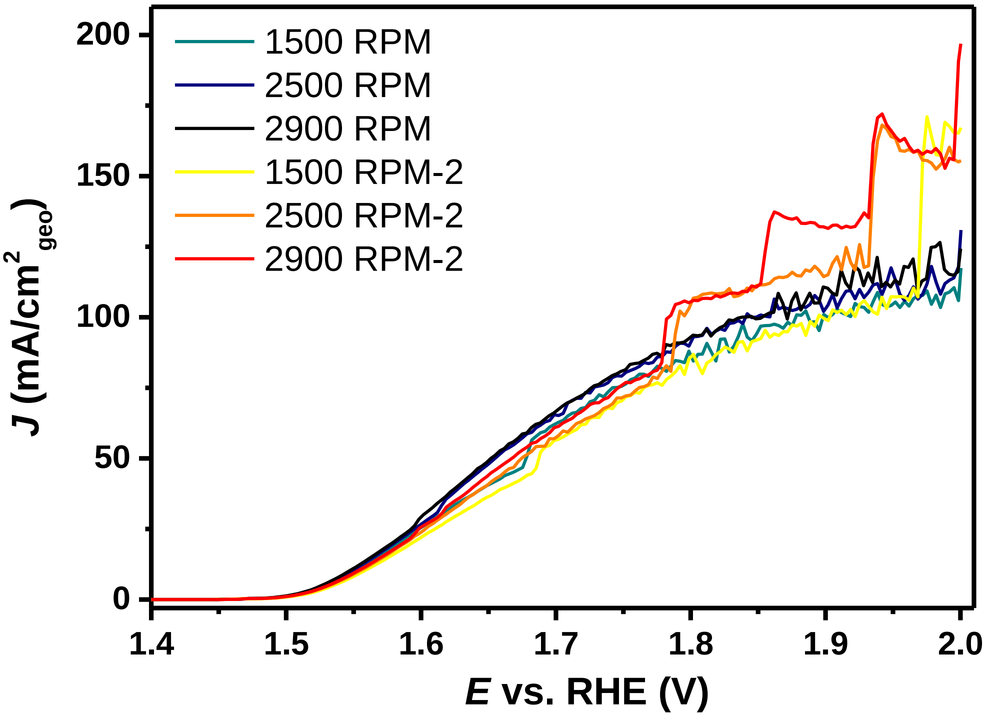

Figure S6. Experimental reproducibility based on the insertion of the rotator tip, and its efficacy in removing large bubbles from its surface strongly effects the reproducibility on the same layer. Experiments 1500/2500/2900 RPM-2 have a further inserted rotator shaft.

LSVs of different layer thicknesses at constant rotation rates from 1000 to 2500 RPM.

Four different electrodeposited layers are compared, which were formed by respectively passing 4.6, 20, 40, 100 mC during electrodeposition with currents of 4 and 8 mA/cm$^2_{Au}$. In Figure S7, the different layers are shown with the rotation rate kept constant. It shows that depending on the current density the order of activity can change from lower rotation rates (where the larger layers relatively have higher activity) to higher rotation rates were the smaller layers are performing increasingly better.

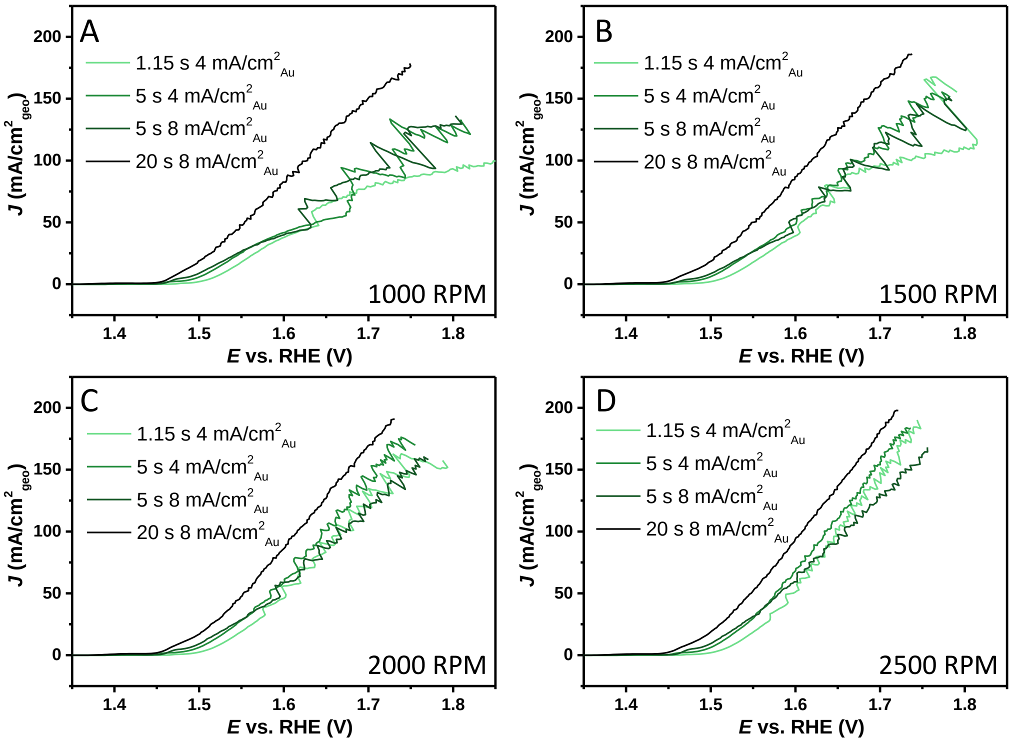

Figure S7. LSV of different layer thicknesses with varying rotation rates from 1000 - 2500 RPM (2900 is used in the main paper, Figure 4). Different rotation rates can result in a change in activity assessment of the different layers.

CVs of NiFeOOH catalysts of different loading normalised to geometric surface area and charge, Ni redox charge to deposition charge.

The geometrical current density is an important metric for the industry, but is often not a good measure of intrinsic catalyst activity. To show how this can change in different current density regions, cyclic voltammograms (CVs) at 10 mV/s are given in Figure S8A, where the largest layer shows the highest geometrical activity. However, the smaller layers have a geometrical current density that becomes increasingly similar to each other at higher current density. In Figure S8B, the CVs are given not normalised to the geometrical surface but to the nickel redox charge,[10] showing how much more effective the smaller layers are when normalized by (an approximation of) its ECSA (Ni redox charge is far from perfect, but somewhat of an indication). In Figure S8C, the deposition charge is compared to the redox charge, showing that each layer of increasing thickness has more electrochemically accessible nickel sites, which for continuous electrodeposition can start to change at high loading. [11]

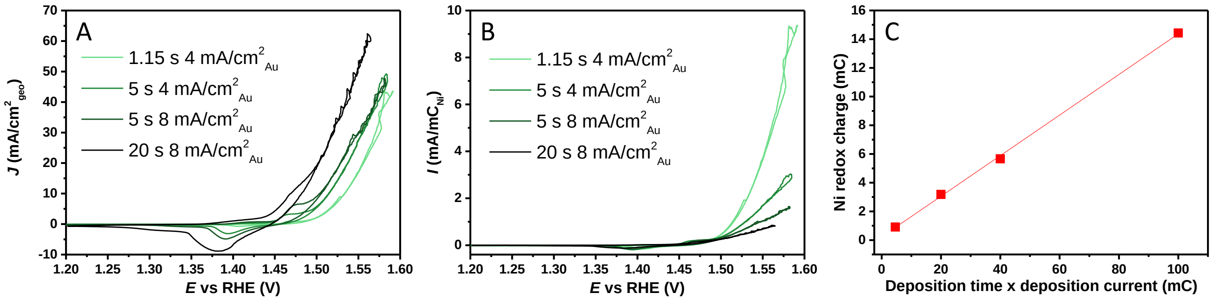

Figure S8. (A) CVs (100% *iR* corrected) vs. geometrical surface area, (B) CVs with current normalised to Ni redox charge, (C) Ni redox charge vs. deposition charge for the four layers depicted, showing a sizeable difference in Ni redox charge between the layers measured. CVs are taken at a scan rate of 10 mV/s and rotation rate of 2500 RPM.

LSV at different scan rates

LSV at different rotation rates shows how bubble formation and release are dependent on the scan rate (layer of 5 s at 8 mA/cm^2). As can be clearly observed in figure S9B, lower scan rates result in a lower potential difference between bubble releases and higher amplitudes. There is a higher Ni oxidation current for higher scan rates, as can be seen from the higher current density near 1.5 V. Usually, a scan rate of 5 mV/s was used, except for the sonication experiments where the scan rate was 20 mV/s.

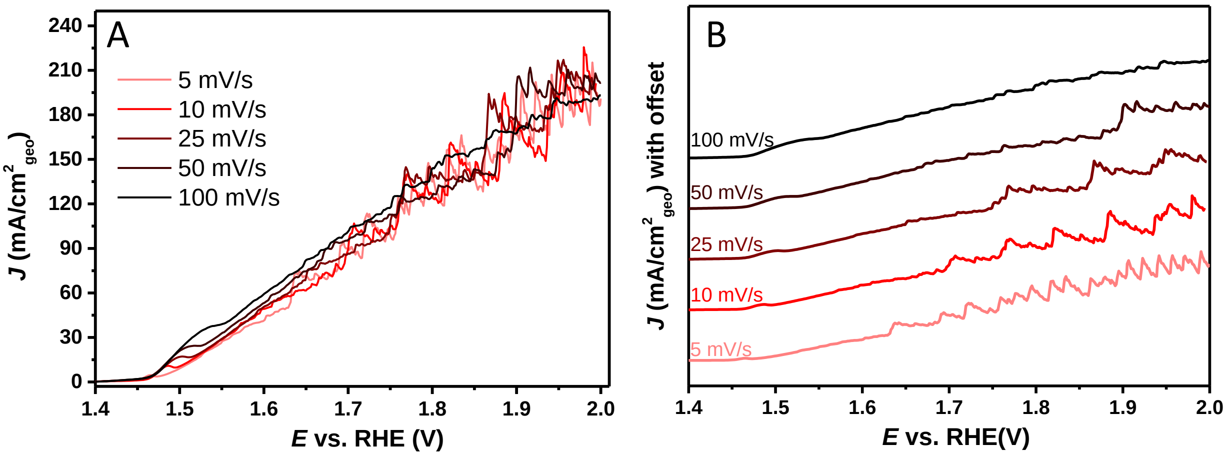

Figure S9. (A) 85% *iR* corrected LSVs, bubble formation and release are dependent on the scan rate, (B) To clarify bubble release and formation patterns, all LSV are given with an offset. Rotation rate was 1000 RPM.

Full LSV for sonication

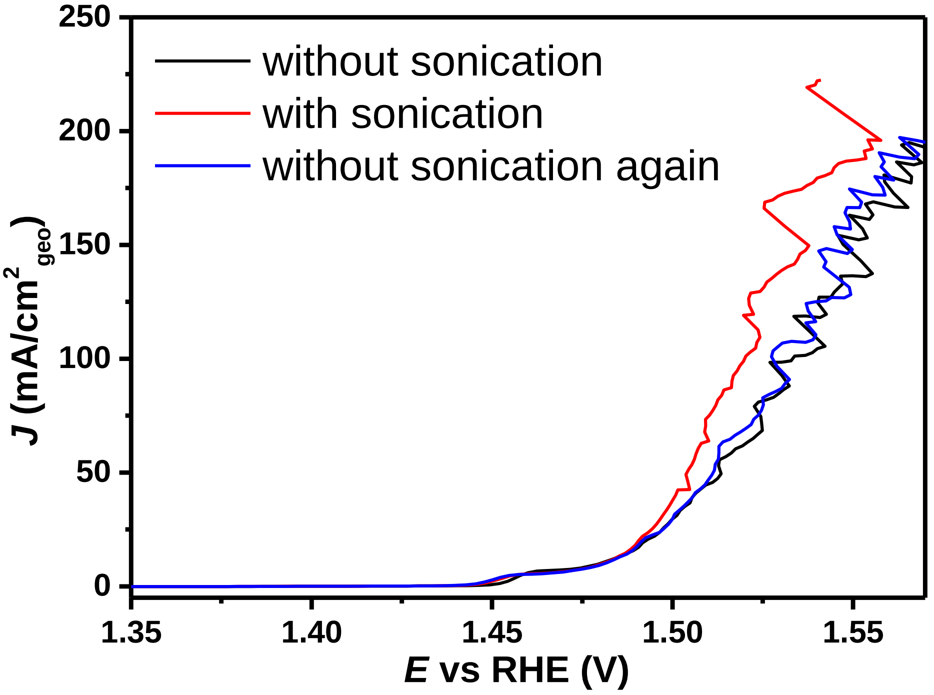

Figure S10. LSV to higher current densities with sonication (red) and without sonication (black and blue). The first scan was without sonication, the next LSV was with sonication and then without sonication again. These curves show that the catalyst activity before and after is the same and that the effect shown in the red curve is indeed caused by sonication. Bath temperature was ~30°C.

References

[1] S. Trasatti and O. A. Petrii, *J. Electroanal. Chem*. **1992**, 327, 353–376.

[2] K. J. J. Mayrhofer, A. S. Crampton, G. K. H. Wiberg, and M. Arenz, *J. Electrochem. Soc*. **2008,** 155, 78.

[3] S. Anantharaj and S. Noda, *ChemElectroChem* **2020**, 7, 2297–2308.

[4] R. Sanchis-Gual, A. Seijas-Da Silva, M. Coronado-Puchau, T. F. Otero, G. Abellán, and E. Coronado, *Electrochim. Acta* **2021**, 388, p. 138613.

[5] T. Shinagawa, A. T. Garcia-Esparza, and K. Takanabe, *Sci. Rep.* **2015**, 5, 1–21.

[6] G. Kear and F. C. Walsh, *Corros. Mater*. **2005**, 30, 51–55.

[7] S. Anantharaj, S. R. Ede, K. Karthick, S. S. Sankar, K. Sangeetha, P.E. Karthik, S. Kundu Energy Environ. Sci. 2018, 11, 744-771.

[8] J. Dukovic and C. W. Tobias, *J. Electrochem. Soc.* **1986**, 134, 331–343.

[9] S. Anantharaj and S. Noda, *ChemElectroChem* **2020**, 7, 2297–2308.

[10] E. Cossar, M. S. E. Houache, Z. Zhang, and E. A. Baranova, *J. Electroanal. Chem.* **2020**, 870, 114246.

[11] A. S. Batchellor and S. W. Boettcher, *ACS Catal.* **2015**, 5, 6680–6689.
